# dbHiMo: a web-based epigenomics platform for histone-modifying enzymes

**DOI:** 10.1093/database/bav052

**Published:** 2015-06-08

**Authors:** Jaeyoung Choi, Ki-Tae Kim, Aram Huh, Seomun Kwon, Changyoung Hong, Fred O. Asiegbu, Junhyun Jeon, Yong-Hwan Lee

**Affiliations:** ^1^Department of Forest Sciences, University of Helsinki, 00014 Helsinki, Finland, ^2^Fungal Bioinformatics Laboratory, Seoul National University, Seoul 151-921, Korea, ^3^Department of Agricultural Biotechnology, College of Agriculture and Life Science, Seoul National University, Seoul 151-921, Korea, ^4^School of Biotechnology, Yeungnam University, Gyeongsan, Gyeongbuk 712-749, Korea, and ^5^Research Institute of Agriculture and Life Sciences, Center for Fungal Pathogenesis, Center for Fungal Genetic Resources, Plant Genomics and Breeding Institute, Seoul National University, Seoul 151-921, Korea

## Abstract

Over the past two decades, epigenetics has evolved into a key concept for understanding regulation of gene expression. Among many epigenetic mechanisms, covalent modifications such as acetylation and methylation of lysine residues on core histones emerged as a major mechanism in epigenetic regulation. Here, we present the database for histone-modifying enzymes (dbHiMo; http://hme.riceblast.snu.ac.kr/) aimed at facilitating functional and comparative analysis of histone-modifying enzymes (HMEs). HMEs were identified by applying a search pipeline built upon profile hidden Markov model (HMM) to proteomes. The database incorporates 11 576 HMEs identified from 603 proteomes including 483 fungal, 32 plants and 51 metazoan species. The dbHiMo provides users with web-based personalized data browsing and analysis tools, supporting comparative and evolutionary genomics. With comprehensive data entries and associated web-based tools, our database will be a valuable resource for future epigenetics/epigenomics studies.

**Database URL:** http://hme.riceblast.snu.ac.kr/

## Introduction

Histones are highly basic, nuclear proteins that provide physical means for eukaryotic DNA to be organized and packaged into chromatin. It has been shown that both the histone tails and globular domains are subjected to a diverse array of the posttranslational covalent modifications (e.g. acetylation and methylation), and that such modifications are pivotal for regulating chromatin dynamics and transcriptional output of the gene/genome (1–4). During the last decades, a number of enzymes that catalyze the addition and removal of histone modifications have been discovered, enabling investigation of roles of complex regulatory network formed by different histone modifications in a variety of cellular processes (5).

Recent surge in the number of sequenced genomes increased the demand for a repository that organizes and compiles information on histone-modifying enzymes (HMEs) in an easily accessible format. To date, databases such as HistoneHits (6), ChromDB (7) and HIstome (8) were presented to provide researchers with information on different histone modifications and enzymes responsible for each modification. However, those databases are centered primarily on yeast, plants and human, lacking curated information on HMEs in the fungal kingdom (other than yeast). Despite the generality of histone modifications as epigenetic mechanisms in eukaryotes, implication of histone modifications in fungal biology is beginning to emerge (9). The number of sequenced genomes from fungi is rapidly increasing and represents the widest sampling of genomes from any eukaryotic kingdom (10). This large volume of data offers unparalleled opportunity for comparative studies to reveal evolution and other fundamental aspects of eukaryotic biology. To take great advantage of such datasets, it is imperative to have a centralized and organized data repository that ensures accessibility to researchers.

To this end, we constructed dbHiMo: a web-based genomics platform for HMEs (http://hme.riceblast.snu.ac.kr/) by using HMM sequence profiles. Domain databases (e.g. Pfam and InterPro) might be used in the gene identification; however, there was some limitation to using domain profiles predicted by InterPro scan. For example, protein sequences belonging to ClassIIB and ClassIV histone deacetylases (HDACs) were indistinguishable, since the two gene families only had IPR000286 in their sequences. Although they had the same domain profile, a phylogenetic tree showed two clear clades of ClassIIB and ClassIV (Supplementary Figure S1A). This indicates that there are fair amount of differences in the sequences. HMM profiles could capture these differences, thus providing a better prediction. The same was true for the two histone acetyltransferases (HATs), Sas2 and Sas3. The sequences belonging to Sas2 and Sas3 shared the same domain profile, containing IPR002717 and IPR016181. However, a phylogenetic tree showed clear distinction between the two gene families (Supplementary Figure S1B). Our primary focus is to catalogue genes predicted to encode HMEs in diverse species with emphasis on fungi and make this information available to scientific communities for comparative analysis after rigorous curation. We believe that knowledge regarding the conservation of the genes involved in histone modification system among diverse species of lower eukaryotes, especially fungi, would be invaluable in understanding the evolutionary aspects of histone modification-mediated epigenetic regulation of gene expression and development. This knowledge in combination with experimental evidence may give more insights into functional importance of a particular histone modifier and their targets in given organisms. dbHiMo incorporates annotation data and analysis tools such as homology search for 603 proteomes, including the species from fungi, plants and animals. We hope that this database would serve as a central portal for providing information on HMEs, stimulating future researches aiming at a deeper understanding of fungal epigenetics.

## Materials and methods

### Collection of sequences and proteomes

For construction of an identification pipeline, protein sequences of 284 annotated genes covering 30 gene families were retrieved from UniProtKB/SwissProt (11) and NCBI databases. The 284 sequences could be browsed at the dbHiMo website under ‘Browse Data’ menu. A total of 603 proteome sequences were obtained from the standardized genome warehouse in Comparative Fungal Genomics Platform 2.0 (CFGP 2.0; http://cfgp.snu.ac.kr/) (12).

### Construction of an identification pipeline for genes encoding HMEs

Protein sequences for each gene family were subjected to multiple sequence alignment using T-Coffee (13). The resulting alignments were trimmed to retrieve well conserved regions by using trimAl (14). HMMER package (15) was used to build sequence profiles (*hmmbuild*) from the trimmed alignments and to search proteome for genes that match the profiles (*hmmsearch*). To remove redundancy in prediction, the gene family with the highest score was chosen for the final prediction, and the others were discarded. For the gene families of Hpa2 and Hpa3, BLAT (16) was used for identification of putative genes due to the lack of sequences available to create alignments. Based on the assumption that protein sequences sharing considerable identity would have the same biochemical function, the cut-off identity was set to be 40% of the query sequences (17). The same redundancy treatment was applied for Hpa2 and Hpa3 just like the other gene families. A total of 603 proteomes covering 483 fungi, 5 Oomycetes, 32 plants and 51 metazoan species were searched by the pipeline.

### Evaluation of the pipeline

To assess the accuracy of the pipeline, a set of sequences was prepared by collecting ones annotated as HMEs from UniProtKB/TrEMBL (11) (positive set). Since the sequences annotated as HMEs in UniProtKB/SwissProt were included in construction of the sequence profiles, the positive set consists of the sequences from UniProtKB/TrEMBL. The sequences were classified into each gene family and scanned by the corresponding sequence profile. The negative sets were prepared for each of the four categories, methyltransferase, acetyltransferase, demethylase and deacetylase, from both UniProtKB/SwissProt and UniProtKB/TrEMBL (11). Each of the four negative sets has the same functionality with different substrate specificities. The substrate difference makes these sequences as a good negative set to test the discrimination power of the pipeline. For example, the negative set for demethylase includes sterol demethylases, pisatin demethylases, nicotine demethylase and so forth. All the six sequence profiles for histone demethylases were subjected to scanning the negative set for demethylase. The resulting data obtained were used to calculate accuracy of the pipeline. Some gene families were unable to be tested due to lack of testable sequences except for the ones used in the construction of the sequence profiles. In order to further validate the pipeline, ‘leave-one-out’ cross-validation was performed. Each of the 282 reference sequences, excluding Hpa2 and Hpa3, was removed only once from the sequence profile, then a new sequence profile was used to detect the removed sequence. As a result, all the sequences were recalled, showing E-value ≤ 4.8e−16. In addition, 91.49% of them showed E-values lower than 1e−100 (Supplementary Table S1).

### Investigation of gene duplication and loss

To calculate gene duplication and loss events, species phylogeny and gene tree for a target gene family were prepared. The species phylogeny was constructed by CVTree (version 4.2.1; source code distribution) (18). The whole proteome sequences were used as input, and K-tuple length was set to be seven, which was shown to be optimal for construction of fungal phylogeny (19, 20). The protein coding nucleotide sequences of GCN5 and PCAF were aligned by using MUSCLE built in MEGA6 (21). The alignment was tested to find the best model for construction of phylogeny. The gene tree was constructed by using Maximum-Likelihood algorithm with the suggested best model. The consensus tree was selected for the final gene tree of GCN5 and PCAF sequences. The species and gene trees prepared were used as input for reconciliation analysis by Notung (version 2.6) (22). In total of 35 genomes covering 29 fungi, 1 Oomycete, 2 plants and 3 animals were chosen for the reconciliation analysis (Table 1).

**Table 1.** List of the 35 genomes used in analysis of gene duplication and loss

Species name	Kingdom	Phylum	Subphylum
*Aspergillus fumigatus* Af293	Fungi	Ascomycota	Pezizomycotina
*Aspergillus nidulans* FGSC A4	Fungi	Ascomycota	Pezizomycotina
*Blumeria graminis* f. sp. *hordei* DH14	Fungi	Ascomycota	Pezizomycotina
*Botrytis cinerea*	Fungi	Ascomycota	Pezizomycotina
*Coccidioides immitis* RS	Fungi	Ascomycota	Pezizomycotina
*Colletotrichum graminicola* M1.001	Fungi	Ascomycota	Pezizomycotina
*Fusarium graminearum*	Fungi	Ascomycota	Pezizomycotina
*Fusarium oxysporum* f. sp. *lycopersici*	Fungi	Ascomycota	Pezizomycotina
*Histoplasma capsulatum* H88	Fungi	Ascomycota	Pezizomycotina
*Magnaporthe oryzae* 70-15	Fungi	Ascomycota	Pezizomycotina
*Mycosphaerella graminicola*	Fungi	Ascomycota	Pezizomycotina
*Neurospora crassa*	Fungi	Ascomycota	Pezizomycotina
*Podospora* anserina	Fungi	Ascomycota	Pezizomycotina
*Candida albicans* SC5314	Fungi	Ascomycota	Saccharomycotina
*Saccharomyces cerevisiae* S288C	Fungi	Ascomycota	Saccharomycotina
*Schizosaccharomyces pombe* 132	Fungi	Ascomycota	Taphrinomycotina
*Heterobasidion irregulare* TC 32-1	Fungi	Basidiomycota	Agaricomycotina
*Laccaria bicolor*	Fungi	Basidiomycota	Agaricomycotina
*Phanerochaete chrysosporium* RP-78	Fungi	Basidiomycota	Agaricomycotina
*Serpula lacrymans* S7.9	Fungi	Basidiomycota	Agaricomycotina
*Cryptococcus neoformans* var. *grubii* H99	Fungi	Basidiomycota	Agricomycotina
*Melampsora laricis-populina* 98AG31	Fungi	Basidiomycota	Pucciniomycotina
*Puccinia graminis* f. sp. *tritici*	Fungi	Basidiomycota	Pucciniomycotina
*Ustilago maydis* 521	Fungi	Basidiomycota	Ustilaginomycotina
*Allomyces macrogynus*	Fungi	Blastocladiomycota	N/D
*Batrachochytrium dendrobatidis* JAM81	Fungi	Chytridiomycota	N/D
*Encephalitozoon cuniculi*	Fungi	Microsporodia	N/D
*Phycomyces blakesleeanus* NRRL1555	Fungi	Zygomycota	Mucoromycotina
*Rhizopus oryzae*	Fungi	Zygomycota	Mucoromycotina
*Phytophthora infestans*	Chromista	Oomycota	Oomycotina
*Arabidopsis thaliana*	Viridiplantae	Streptophyta	N/D
*Oryza sativa*	Viridiplantae	Streptophyta	N/D
Drosophila *melanogaster*	Metazoa	Arthropoda	N/D
*Caenorhabditis elegans*	Metazoa	Nematoda	N/D
*Homo sapiens*	Metazoa	Chordata	Craniata

## Results

### Evaluation of the pipeline

Evaluation of the pipeline was performed using positive and negative sets of protein sequences. Among total of 6669 sequences belonging to the negative sets, no hit of which E-value is below 1.0e−5 was detected. The most significant hit was found when the negative set for methyltransferase was searched by the PRMT_2 profile, showing E-value of 0.069. In total, only five other sequences showed E-value below 1.0, ranging from 0.38 and 0.92, which is far higher than the threshold of positive prediction. For the positive set, the pipeline showed the 95.24 and 100% of sensitivity and specificity, on average (Supplementary Table S2). These results clearly supports that the pipeline has a satisfying prediction accuracy as well as good discrimination power against the negative sets.

### Distribution of putative genes encoding HMEs across the taxonomy

**Figure 1.**
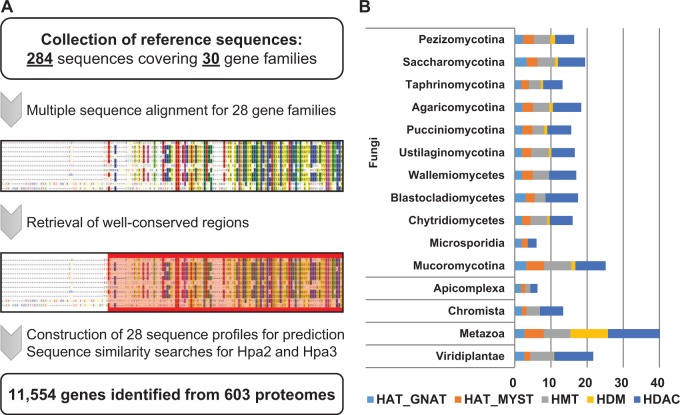
An identification pipeline and prediction summary of dbHiMo. A schematic flowchart of the dbHiMo pipeline and distribution of the predicted genes across the taxonomy. (**A**) *In silico* prediction pipeline consists of three steps: (i) collection of the reference sequences for each gene family, (ii) multiple sequence alignment and retrieval of well-conserved regions and (iii) construction of sequence profiles and searching on the proteomes. (**B**) The average numbers of genes belonging to the five main categories for a given taxonomy were summarized to show overview of the prediction results.

From 603 proteomes, our pipeline identified 11 554 genes encode putative HMEs (Figure 1; Table 2). Among the 30 gene families, ELP3, GCN5, Esa1, PRMT1 and ClassI/III HDACs were found across the taxonomy covering *Plasmodium* spp, Oomycetes, fungi, plants and animals (Supplementary Table S3). This suggests that these enzymes are the most ancient and essential ones in all organisms. Not surprisingly, PCAF was found to be Metazoa-specific, which is known as functional orthologue of GCN5 (23). Only a few insect species were predicted to have GCN5-type proteins, whereas the majority of metazoan species have only PCAF. Besides PCAF, there were several taxon-specific gene families (Supplementary Table S3). The gene families of HBO1, MOZ_MORF, DOT1L, PRMT_2, JHDM1, JHDM2, PHF2_PHF8, UTX_JMJD3 and ClassIIA were found to be Metazoa-specific. In addition, Hpa2 and Hpa3 were Saccharomycotina-specific, mainly in *Saccharomyces cerevisiae* and *S. paradoxus*. The most Sas3 genes were found in the phylum Ascomycota. Though the majority of Sas3 genes was identified in the subphylum Saccharomycotina, the putative Sas3 genes were also frequently found in the genera of *Aspergillus*, *Penicillium* and *Candida*. DOT1 was another gene family, which was most commonly found in the subphylum Saccharomycotina, however, the putative DOT1 genes were also found in other 144 fungal proteomes. Interestingly, species belonging to the phylum Microsporidia lack many gene families, showing the least number of HME-encoding genes (Figure 1B). In fact, Microsporidia had fewer predicted gene families than any other taxa, which is in agreement with genome compaction and gene loss reported in the previous studies (24, 25).

**Table 2.** List of gene families available in dbHiMo

Category	Gene family	Number of genes	Number of genomes
Histone acetyltransferase	ELP3	634	589
(HAT; GNAT^a^ family)	GCN5	641	536
	Hpa2	70	70
	Hpa3	68	67
	PCAF	74	51
Histone acetyltransferase	Esa1	691	526
(HAT; MYST^b^ family)	HBO1	75	53
	MOF	127	93
	MOZ_MORF	39	14
	Sas2	519	475
	Sas3	145	144
	Tip60	212	182
Histone deacetylase	ClassI	1832	602
	ClassIIA	115	44
	ClassIIB	628	524
	ClassIII	1658	589
	ClassIV	120	83
Histone methyltransferase	DOT1	248	247
(HMT)	DOT1L	19	14
	PRMT_1	1375	590
	PRMT_2	36	17
	SET1	442	420
	SET2	533	508
	SET5	201	200
Histone demethylase	JARID1	209	140
(HDM)	JHDM1	63	39
	JHDM2	73	34
	JHDM3_JMJD2	551	472
	PHF2_PHF8	64	22
	UTX_JMJD3	92	50

^a^GNAT is the abbreviation for Gcn5-related *N*-acetyltransferases (35).

^b^MYST is named after its members, including MOZ, Ybf2 (Sas3), Sas2 and Tip60 (35).

Histone demethylase (HDM)-encoding genes were more frequently found in Metazoa than any other taxon. Interestingly, unicellular organisms, including *Capsaspora owczarzaki* ATCC 30864, *Monosiga brevicollis* and *Proterospongia* sp. ATCC 50818, were predicted to have a putative gene encoding JHDM3_JMJD2. Considering the fact that these unicellular species are believed to be closely related to multicellular Metazoa (26–28) and most of fungal genomes have only one copy of this gene, the ancestral JHDM3_JMJD2-encoding gene could have existed before multi-cellularity appeared.

The average number of genes encoding HDAC was larger than any other enzyme families. However, *Plasmodium* spp. and Microsporidia showed far less number of genes. ClassIV HDAC was absent in fungi and Oomycetes, but found in 77 out of 83 genomes of the species belonging to Metazoa and Viridiplantae. In addition, three out of six *Plasmodium* spp. were also predicted to have the genes. Besides the aforementioned species, the only species possessing this gene was *C. owczarzaki* ATCC 30864, *Rhizophagus irregularis* DAOM 181 602 and *Paramecium tetraurelia*, which do not belong to fungi, Oomycota, animals or plants.

### Archaeal HMEs

Previously, an archaeal lysine methyltransferase, aKMT4 (NCBI accession; YP_005648729), was characterized from *Sulfolobus islandicus* REY15A and reported that it has relatively high similarity to *S. cerevisiae* DOT1 (29). When aKMT4 was searched by the DOT1 sequence profile, however, no significant hit was detected. In fact, BLAST searches of aKMT4 against *S. cerevisiae* DOT1 showed E-value of 1.7. According to our analysis, aKMT4 showed a bit better hits when it was searched against the PRMT1 sequences which were used in the construction of the sequence profile. Even if there were two hits with E-value of 4.0e−7 and 5.0e−6 against *Schizosaccharomyces pombe* Rmt1 (NP_594825) and *Danio rerio* PRMT1 (NP_956944), respectively, query-based sequence identity did not exceed 21.11%. This is probably because that different structure of archaeal histone, especially absence of ‘tails’ (30). Therefore, archaeal HMEs might have evolved to take different targets from those of eukaryotes, hence exhibiting clear difference at the sequence-level.

### Whole-genome duplication and the copy number of HMEs

It has been reported in a number of studies that whole-genome duplication (WGD) occurred in *Saccharomyces* genus (31), and autopolyploidy and allopolyploidy were found as a result of WGD (32, 33). It was characterized that *S. pastorianus* strain Weihenstephan 34/70 is allotetraploid of *S. cerevisiae* and *S. eubayanus* (34). Such WGD events are reflected in the number of HME-encoding genes. The number of HME-encoding genes in *S. pastorianus* Weihenstephan 34/70 showed almost twice as many number of genes as that of the other species belong to the subphylum Saccharomycotina (Supplementary Table S3). However, only one HDM-encoding gene was predicted, just like 88 out of 108 other species belonging to Saccharomycotina, implying post-genome rearrangement after establishment of allotetraploidy.

### System architecture

System design and web engine of dbHiMo were inherited from those of CFGP 2.0. dbHiMo adopted the three-tier system, which consists of database, application and presentation tiers. Each tier has physically separate servers for better load distribution and user experience. Data-driven user interface (DUI) was also implemented from CFGP 2.0, which enables users to share and analyse their sequence collections at any sister systems via ‘Favorite Browser’. Web pages were developed by using HTML5, PHP, CSS3 and Javascript (Ajax/jQuery) in order to support the compatibility among the web browsers. MySQL is the database management system. Web pages are hosted through Apache HTTP servers. The script of an automated pipeline was written in Perl with the in-house module libraries.

## Discussion and utility

We developed an epigenomics portal for comparative and evolutionary analysis (dbHiMo; http://hme.riceblast.snu.ac.kr/). dbHiMo offers a fungal kingdom-wide catalogue of HME-encoding genes as well as the predicted genes from plant and animal genomes for comparative analysis purpose. Our compilation of data coupled with analysis functionalities will be a valuable resource for scientists working on epigenetic regulation of transcription as well as evolution of epigenetic mechanisms. Data and functions available at dbHiMo have several potential applications for the users, including genome-wide functional characterization, comparative sequence analysis and analyses of gene family evolution. We provide one exemplary application using our prediction data to speculate the evolutionary history of HAT families, GCN5 and PCAF. This example illustrates how dbHiMo can aid in future researches aiming at comparative and evolutionary analysis.

### Web utility

**Figure 2.**
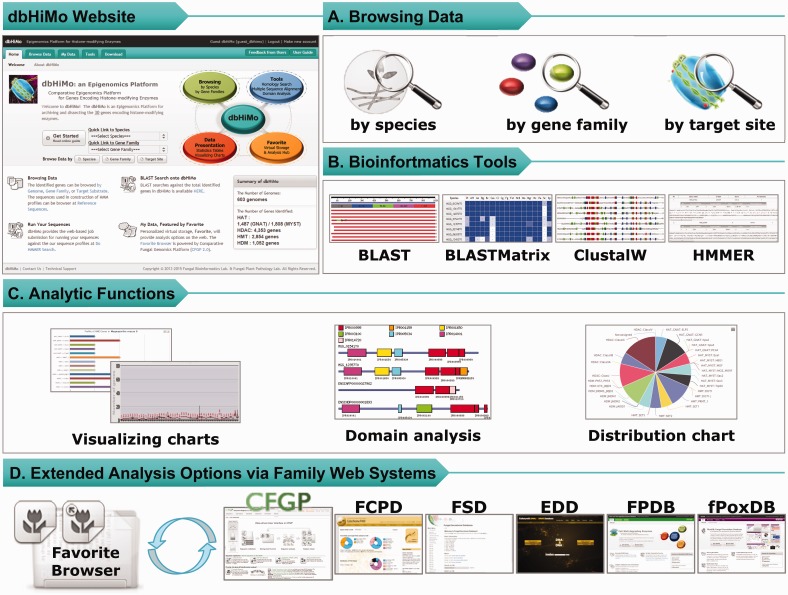
Web functionality available on dbHiMo website. (**A**) Web interface of dbHiMo supports browsing methods (i) by species (or genome), (ii) gene family or (iii) target site in histone. (**B**) Bioinformatics tools are available on the web; (i) sequence similarity searches (BLAST and BLASTMatrix), (ii) multiple sequence alignment (ClustalW) and (iii) prediction of sequence(s) provided by users. (**C**) Analytic functions are provided including (i) distribution chart/table of genes in a genome, (ii) distribution across the taxonomy for a given gene family, (iii) domain architecture analysis and (iv) distribution of genes from a sequence collection in Favorite Browser. (D) Sequence collections in Favorite Browser can be further analysed by the tools available at the CFGP 2.0 and other sister databases.

In support of comparative and evolutionary analysis of HMEs, dbHiMo website provides users with user-interface enabling (i) browsing by species/gene family/target site, (ii) browsing taxonomical distribution of enzymes, (iii) protein domain analysis and (iv) extended analysis using ‘Favorite’ function implemented in CFGP 2.0 (Figure 2). Furthermore, bioinformatics tools for homology search and multiple sequence alignment will also be provided. Besides browsing by species and enzyme family, dbHiMo provides browsing by target site for better data exploration, which is possible due to the high specificity of HMEs on their respective target site (23). Users can catalogue the whole genes for a given target site, or the gene families corresponding to the site. Diverse browsing methods will greatly increase usability, which enable users to narrow down on the genes of interest quickly.

### Evolutionary history of genes encoding GCN5 and PCAF

Investigation of evolutionary history of genes and domains would help us better understand underpinnings behind various biological puzzles, such as differential distribution of particular gene families among diverse taxa. To illustrate that our database can facilitate such investigation of HMEs, we performed an evolutionary analysis using our prediction results of HAT families, GCN5 and PCAF. In fungi, GCN5 is known to be essential for virulence of *Cryptococcus neoformans* (36). In *Trichoderma reesei*, GCN5 was shown to regulate growth, conidiation as well as cellulase gene expression (37). In *A. nidulans*, it regulates asexual development (38). Furthermore, it is known that the catalytic domain of human GCN5 can functionally complement the yeast counterpart, suggesting GCN5 is functionally conserved in eukaryotic species (39). We found that presence of GCN5-encoding genes is a universal feature of fungal genomes, showing 98.14% (475 out of 484) of fungal genomes have at least one GCN5-encoding gene. However, 81 genomes were predicted to have multiple copies (Supplementary Table S3). Thus, we tried to elucidate where this copy number difference resulted from.

**Figure 3.**
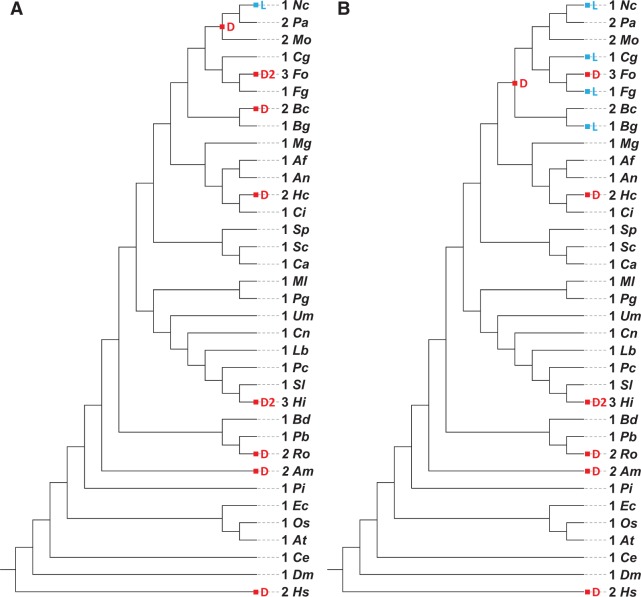
Duplications and losses calculated for a HAT enzyme, GCN5/PCAF. The reconciled tree of GCN5/PCAF sequences from 35 species covering fungi, Oomycetes, animals and plants was constructed. The numbers of gene duplication (D) and loss (L) events are condensed to the species tree and shown in the corresponding internal node. The number of genes and the species name are presented next to the leaf nodes. Species names are abbreviated as the following (ordered by appearance in the tree): Nc (*Neurospora crassa*), Pa (*Podospora anserina*), Mo (*Magnaporthe oryzae* 70–15), Cg (*Colletotrichum graminicola* M1.001), Fo (*Fusarium oxysporum* f. sp. *lycopersici*), Fg (*F. graminearum*), Bc (*Botrytis cinerea*), Bg (*Blumeria graminis* f. sp. *hordei* DH14), Mg (*Mycosphaerella graminicola*), Af (*Aspergillus fumigatus* Af293), An (*A. nidulans* FGSC A4), Hc (*Histoplasma capsulatum* H88), Ci (*Coccidioides immitis* RS), Sp (*Schizosaccharomyces pombe* 132), Sc (*Saccharomyces cerevisiae* S288C), Ca (*Candida albicans* SC5314), Ml (*Melampsora laricis-populina* 98AG31), Pg (*Puccinia graminis* f. sp. *tritici*), Um (*Ustilago maydis* 521), Cn (*Cryptococcus neoformans* var. *grubii* H99), Lb (*Laccaria bicolor*), Pc (*Phanerochaete chrysosporium* RP-78), Sl (*Serpula lacrymans* S7.9), Hi (*Heterobasidion irregulare* TC 32–1), Bd (*Batrachochytrium dendrobatidis* JAM81), Pb (*Phycomyces blakesleeanus* NRRL1555), Ro (*Rhizopus oryzae*), Am (*Allomyces macrogynus*), Pi (*Phytophthora infestans*), Ec (*Encephalitozoon cuniculi*), Os (*Oryza sativa*), At (*Arabidopsis thaliana*), Ce (*Caenorhabditis elegans*), Dm (*Drosophila melanogaster*) and Hs (*Homo sapiens*).

GCN5 and PCAF are known to share the same substrate specificity and often categorized into one gene family, KAT2 (lysine-acetyltransferase 2) (23). The number of GCN5-encoding genes is not variable among the fungi, showing 1.18 genes per genome with standard deviation of 0.49. It might imply that KAT2 genes are conserved throughout the fungal evolution. In order to investigate evolutionary history of the genes in detail, reconciliation analysis was performed with the selected genomes (Table 1). Interestingly, two potential evolutionary histories were resolved from the reconciliation analysis (Figure 3). Two histories presented the same result outside of the two classes, Sordariomycetes and Leotiomycetes. The first candidate (Figure 3A) reflected that multi-copy of GCN5-encoding genes were achieved by recent duplications, whereas the other (Figure 3B) indicated that these were the result of multiple species-level losses in the species having only one copy. In both of possibilities, gene duplications and losses were more frequently found in internal nodes encompassing the species belonging to the subphylum Pezizomycotina (Figure 3). After the divergence of two subphyla Pezizomycotina and Saccharomycotina, multiple duplications and losses were detected down the successive lineages of the phylum Pezizomycotina. In Basidiomycota, however, no duplication and loss was found, except for *Heterobasidion irregulare* TC 32-1. It might imply that gene duplication and loss events have recently occurred. This result also suggests that GCN5-encoding genes might be highly conserved from the ancestral form. In fact, multiple sequence alignment of the 46 genes used in this analysis showed that functional domains were very well conserved, such as IPR000182 (GCN5-related *N*-acetyltransferase) and IPR001487 (Bromodomain) domains. It is demonstrated by the 164 conserved amino acid residues (conserved >70% in the alignment) mainly in IPR000182 (GCN5-related *N*-acetyltransferase) and IPR001487 (Bromodomain) regions, which are important for their function. Moreover, the predicted genes in a species usually showed extremely high sequence similarity. For example, two of three GCN5 sequences in *H. irregulare* TC 32-1 shared 498 out of 502 amino acids in common. Although the other one is longer than aforementioned two sequences, the overall signature remains unchanged. Other species including *Magnaporthe oryzae* 70-15, *Allomyces macrogynus* and *Fusarium oxysporum* also showed similar patterns. In other multi-copy gene families, however, it is not common to observe such high sequence similarities. For example, peroxidases, plant cell wall-degrading enzymes and RNAi proteins (Argonaute, Dicer and RNA-dependent RNA polymerase) are commonly present in multi-copy. However, they do not show such high sequence identity as GCN5/PCAF does. Based on the lines of observations, it is speculated that duplication of GCN5-encoding genes may have occurred quite recently in a species-specific manner. Thus, Figure 3A would represent more probable scenario for evolution of KAT2 sequences than Figure 3B.

## Future prospect

dbHiMo provides a broad archive and analysis tools for HME-encoding genes. In order to follow up with the rapidly released and updated eukaryotic genomes, dbHiMo will be updated in conjunction with regular maintenance of CFGP 2.0. In order to provide better user experience, target substrate data for HMEs will be updated if new molecular characterization becomes available. We will also try to integrate more useful modules, software and/or incorporation of biological knowledge to continuously improve the environment for comparative and evolutionary epigenomics studies.

## Supplementary Data

Supplementary data are available at *Database* Online.

Supplementary Data
