# Supplementary material for: dbHiMo: a web-based epigenomics platform for histone-modifying enzymes
Source: Database (Oxford). 2015 Jun 8;2015:bav052. doi: 10.1093/database/bav052 (PMC4460409; doi:10.1093/database/bav052)
Supplement: Supplementary Data [file supp_2015_bav052_index.html]

dbHiMo: a web-based epigenomics platform for histone-modifying enzymes — Supplementary Data 

# dbHiMo: a web-based epigenomics platform for histone-modifying enzymes

## Supplementary Data

files

- Supplementary Data - zip file
