# Supplementary figures and images for: dbHiMo: a web-based epigenomics platform for histone-modifying enzymes
Source: Database (Oxford). 2015 Jun 8;2015:bav052. doi: 10.1093/database/bav052 (PMC4460409; doi:10.1093/database/bav052)

Supplementary Figure S1

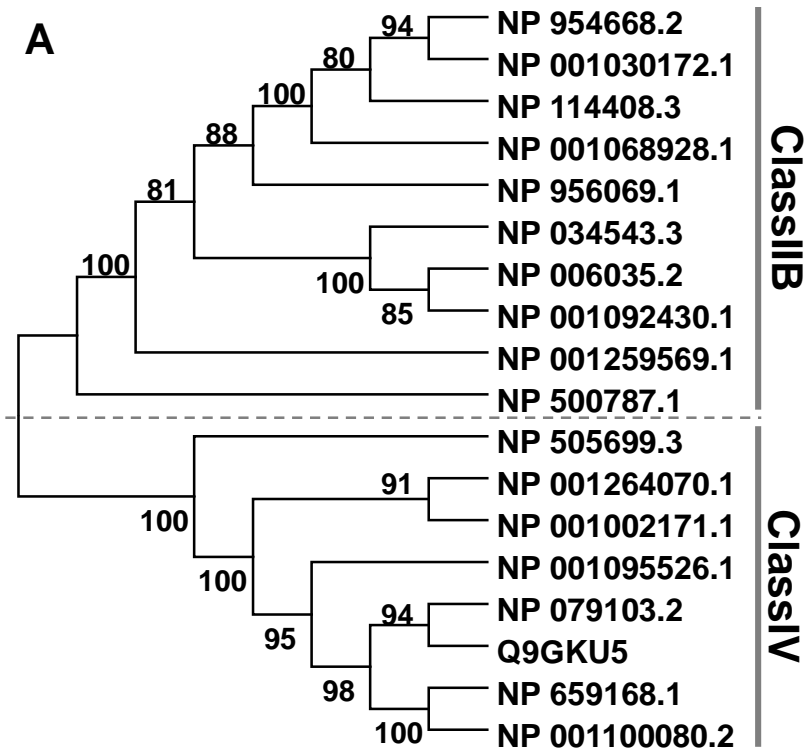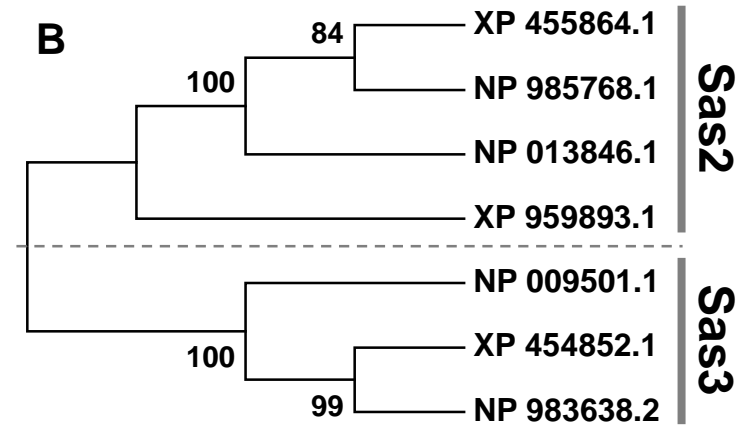

Supplement: Supplementary Data [file supp_bav052_suppl_data.zip › SupplementaryFigureS1.pdf]
